# *In Utero* Domoic Acid Toxicity: A Fetal Basis to Adult Disease in the California Sea Lion (*Zalophus californianus*)

**DOI:** 10.3390/md20080013

**Published:** 2008-06-06

**Authors:** John S. Ramsdell, Tanja S. Zabka

**Affiliations:** 1 Marine Biotoxins Program, Center for Coastal Environmental Health and Biomolecular Research, NOAA, National Ocean Service, Charleston, SC 29414; E-mail: john.ramsdell@noaa.gov; 2 The Marine Mammal Center, 1065 Fort Cronkhite, Marin Headlands, Sausalito, CA 94965, USA. Email: tszabka@yahoo.com

**Keywords:** Domoic acid, Algae, California, Reproduction, Neurodevelopment, Seizure, Sea Lion

## Abstract

California sea lions have been a repeated subject of investigation for early life toxicity, which has been documented to occur with increasing frequency from late February through mid-May in association with organochlorine (PCB and DDT) poisoning and infectious disease in the 1970’s and domoic acid poisoning in the last decade. The mass early life mortality events result from the concentrated breeding grounds and synchronization of reproduction over a 28 day post partum estrus cycle and 11 month *in utero* phase. This physiological synchronization is triggered by a decreasing photoperiod of 11.48 h/day that occurs approximately 90 days after conception at the major California breeding grounds. The photoperiod trigger activates implantation of embryos to proceed with development for the next 242 days until birth. Embryonic diapause is a selectable trait thought to optimize timing for food utilization and male migratory patterns; yet from the toxicological perspective presented here also serves to synchronize developmental toxicity of pulsed environmental events such as domoic acid poisoning. Research studies in laboratory animals have defined age-dependent neurotoxic effects during development and windows of susceptibility to domoic acid exposure. This review will evaluate experimental domoic acid neurotoxicity in developing rodents and, aided by comparative allometric projections, will analyze potential prenatal toxicity and exposure susceptibility in the California sea lion. This analysis should provide a useful tool to forecast fetal toxicity and understand the impact of fetal toxicity on adult disease of the California sea lion.

## California Sea Lion Prenatal Mortality Events

**Figure 1:**
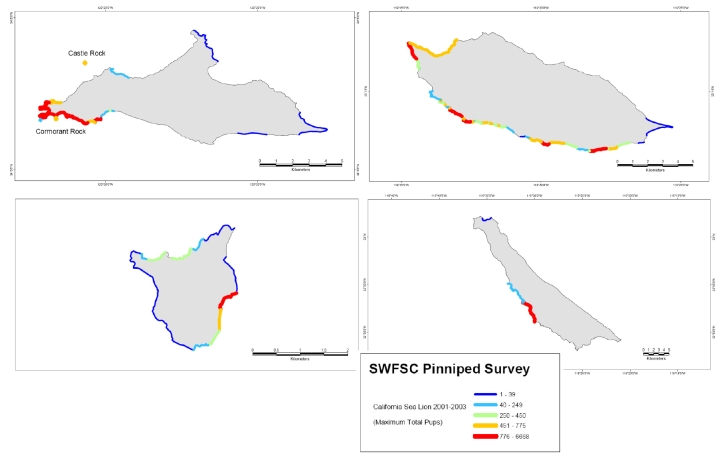
Abundance of California Sea Lion Pups at the Major Rookeries Data is from NOAA Southeast Fisheries Science Center 2001–2003 Survey of pups on the five Channel Island sea lion rookeries. The four islands shown are San Miguel (top), Santa Barbara (second from top), San Nicolas (third from top) and San Clemente (bottom), of which San Miguel and San Nicolas are the major breeding rookeries, and their geographic position also is shown in Figure 2. The survey information was binned into five grouping of animal density (animals/km) (blue 1–16, light blue 17–200, green 201–372, orange 373–688 and red 689–3286) and coded to geographic coordinates of the five islands as described in reference [6].

The California sea lion (*Zalophus californianus*) reproduces at several well defined rookeries off the coast from Mexico to southern California, of which one primary site is the Channel Islands off southern California (Figure 1). Females are present year-round with parturition occurring from mid-May through June and breeding occurring from July through mid-August [1]. These concentrated nursery grounds facilitate human observation, which has identified incidences of mass perinatal mortality of multiple and complex origins. Premature parturition and death of large cohorts of pups between 1968 and 1971 were correlated with high maternal and fetal body concentrations of DDTs and PCBs [2,3]. A cause-effect relationship, however, between organochlorine compounds and reproductive failure was confounded by the later identification of concurrent viral and bacterial infections in these pups [4,5].

Since the elimination of DDT and PCB discharge in 1977 there has been an overall trend towards reduced body burden of summed PCBs and DDTs, and other causes of reproductive failure have emerged [7–10]. More specifically, the body burden of DDT in the fetus through adult life stages has declined substantially, and the body burden of PCB seems to have declined in the fetus and pup, while the trend for adults is not clear. In comparison to other marine mammals, however, the body burden of both classes of compounds in California sea lions remains higher [7,8] Over the last decade, reproductive failure in California sea lions has been associated increasingly with harmful algal blooms, most notably domoic acid produced by *Pseudo-nitzschia* spp. [10–12]. The first documented incidence of domoic acid toxicity in sea lions occurred between May 15 and June 19, 1998, resulting in a mass stranding from Monterey Bay to San Diego due to acute neurologic disease [11,13,14]. Of the group of 70 animals admitted for rehabilitation, 54 were adult females, and almost 1/3^rd^ of these females experienced prenatal reproductive failure [15]. The 1998 event and an even larger event in 2002, which also occurred just prior to scheduled parturition, resulted in 209 documented cases of domoic acid associated reproductive failure [12]. Since these initial events with documentation of acute neurologic disease and reproductive failure in sea lions, additional conditions associated with exposure include long term neurologic sequela from sublethal exposure(s) [16] and a degenerative cardiomyopathy associated with acute or sublethal exposure(s) [17]. This review will consider the potential of *in utero* exposure as a causative factor for another form of domoic acid poisoning, a chronic juvenile and adult neurologic disease.

## Rookeries Place Prenates at High Risk to Domoic Acid Producing Diatom Blooms

**Figure 2:**
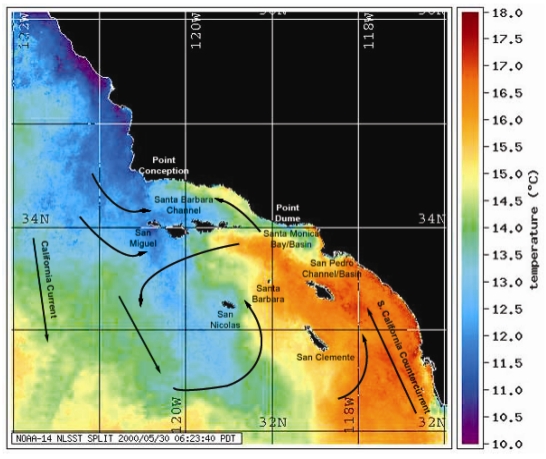
Ocean temperature and current patterns of the South California Bight NOAA-14 satellite map of sea surface temperatures (SSTs) showing upwelling from Pt. Conception bathing the two major California sea lion rookeries (San Miguel and San Nicolas) with cold nutrient-rich waters. Overlay of directional currents is from reference [21].

Diatom blooms are a common occurrence in coastal ocean upwelling systems, such as that found off the coast of California, where late spring northerly winds traditionally bring nutrient-rich deep water into the euphotic zone. The pennate diatom genus *Pseudo-nitzschia* reaches high concentrations during upwelling events and several species (especially *P. australis* in California waters) commonly produce the neurotoxin domoic acid [14]. A noted upwelling region is offshore of Pt. Conception, an abrupt geographical north-south deviation point, which greatly affects the major California rookeries for California sea lions (Figure 2). Upwelling filaments originating just north of Pt. Conception bring cool nutrient-rich waters, and currents can drive the nutrient-rich waters and *Pseudo-nitzschia* blooms north to Monterey Bay as well as south to the two major rookeries, the San Miguel and San Nicolas Islands. The food web plays the primary role in the transmission of domoic acid from *Pseudo-nitzschia* blooms to the California sea lion [11,18]. Common vectors are pelagic planktivorous fish, including the northern anchovy (*Engraulis mordax*) and Pacific sardine (*Sardinops sagax*), which accumulate domoic acid-containing diatoms in their gut exceeding toxin concentrations of one part per thousand, exceptionally high levels for a natural toxin [18,19]. Additionally, *Pseudo-nitzschia*, which form long chains of cells, will sink to the ocean floor where the domoic acid effectively infiltrates the benthic food web and provides an additional source of vectoring [20].

Domoic acid has limited oral effectiveness in adult animals and its subsequent systemic redistribution has a very short half life and poor penetration into the central nervous system. Yet at exposure levels of milligrams per kilograms, as is realistic for a California sea lion [22], domoic acid is a damaging neurotoxin. At the level of the receptor in the brain domoic acid binds to kainate subtypes of ionotropic glutamate receptors to induce excitotoxicity by release of glutamate and activation of NMDA ionotropic glutamate receptors [23]. Excitotoxicity leads to cellular and ultrastructural damage to pathways responsible for the learning and recall of spatial memory and for the restraint of seizure-prone brain circuitry. Toxicity occurring during neurodevelopment, however, can assume a different set of parameters with persistent toxicity manifesting later in juvenile and adult life [24]. As discussed in further detail in this review, domoic acid crosses the placenta, readily enters the neonate brain and is retained in the amniotic fluid. The early fetal brain is electrically silent, but expresses levels of ionotropic glutamate receptors that guide the migration of neurons to the appropriate brain regions and facilitate in the formation correct synapses. Thus, the southern passage of *Pseudo-nitzschia* blooms down the California coast contaminates the foodweb that sustains California sea lions inhabiting the Channel Island rookeries, subjecting their prenates to high *in utero* domoic acid and a different set of toxic effects, as we hypothesize that persistent toxicity can manifest later in juvenile and adult life.

## Embryonic Diapause Synchronizes California Sea Lion Neurodevelopment

**Figure 3:**
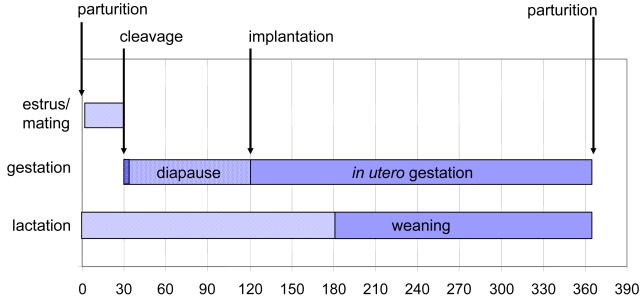
Temporal Relationship of the Annual Reproductive Events of the California Sea Lion The annual reproductive cycle begins with a 28 day estrus following birth. Fertilization leads to embryonic cleavages until the early blastocyst stage. Implantation is delayed until a photoperiod trigger impacts circadial secretory patterns of prolactin and melatonin. Diapause release leads to trophoblast implantation and resumption of embryonic growth. Term development occurs 242 days from release of diapause. Figure design modified is from reference [25] with data from sources cited in the text of the current review.

The reproductive timing of the California sea lion provides a unique opportunity to evaluate wildlife toxicity events such as those caused by domoic acid producing diatom blooms. Term parturition occurs at a mean date of June 15^th^ on San Miguel Island [2]. Mating after a 28 day estrus yields a full year reproductive cycle, resulting in an eleven-month pregnancy with an approximate 90 day embryonic diapause (Figure 3). Lactation continues after parturition for approximately six months with weaning for the final months to the next term birth. Pups normally fed for 1–2 days interspersed with 2–3 days of maternal foraging within a range of 50 km of the rookery.

**Figure 4:**
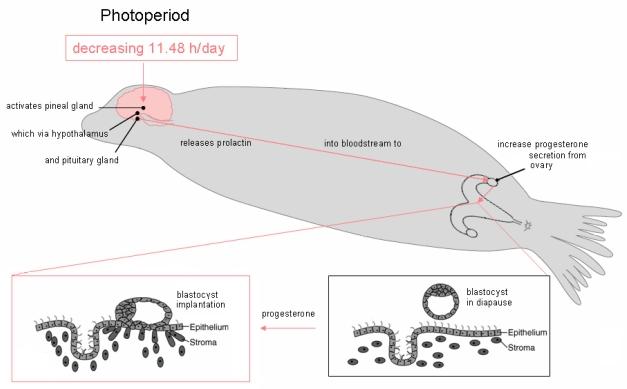
Effect of Photoperiod on Release of California Sea Lion Blastocysts from Diapause A decreasing light cycle of 11.48 hr/day triggers the pineal gland to alter release of the neurohormone melatonin, which via hypothalamic neurons leads to release of pituitary hormones. The endocrine pathways differ between species and have not been elucidated for the California sea lion. Likely hormonal pathway involves inhibition of pineal melatonin, inhibition of hypothalamic dopamine, increase of pituitary prolactin and increase of corpus luteal (ovarian) progesterone. Progesterone and undefined ovarian factors likely induce uterine production of cytokines and growth factors which, in turn, promote receptor mediated adhesion of the blastocyst to the endometrium, initiating implantation. Figure design is modified from reference [25].

Observational differences in sea lion pupping in different geographic locations suggested a variation of parturition based upon latitude [26,27]. The precise timing of release from diapause for California sea lion embyros was determined using captive populations at various zoological facilities and determined to correspond with a common decreasing photoperiod of 11.48 + 0.38 h/day, which corresponds to 242 days of gestation from implantation to birth (Figure 4) [28]. Although the precision of this photoperiod in captive populations is high, other factors such as migration and food supply, might be expected to affect whether parturition occurs early or late in natural populations. These factors likely do not affect synchronization of neurodevelopment, although they will have a certain impact on exposure susceptibility, since parturition marks a major change in the route of toxin exposure to the neonate.

## Comparative Animal Models for California Sea Lion Neurodevelopment

The lack of data on California sea lion neurodevelopment is a major impediment to understanding the hazards associated with neonatal toxicity to domoic acid. Hence, better understanding of sea lion developmental neurotoxicity requires data interpolation from other animals to supplement opportunistic data in California sea lions from natural exposures. The data in this manuscript is based on gross evaluation of the brain from four prenates collected from California sea lions that stranded from January 31, 2006 to April 25, 2007 (E127 to E211) and by corresponding histologic evaluation from four other age-matched prenates as well as an additional prenate from December 13, 2006 (E78) for which gross images were not available for inclusion here. The age of the prenates is calculated as embryonic days based upon release of diapause at the decreasing daylight hour of 11:48 corresponding to 3 October 2006 with the addition of six days as an estimated time of fertilization to blastocyst. Selection of comparative animal models for neurodevelopment is complex because of differences in the length of gestation and the maturation of neurodevelopment at birth, factors which do not correlate well among species. Together these factors have a profound effect on developmental toxicity with a primary effect on route of exposure. Parturition leads to a transition of primary routes of maternal-neonate toxin transport, from the direct transfer via the placenta in the prenatal animal to gastrointestinal absorption via lactation or prey consumption in the postnatal animal. Differences in brain maturation at birth can affect the extent to which brain development stages are susceptible to pre- *vs.* postnatal exposure routes.

California sea lions as adults have a large brain of 362 g (female) and 405 g (male) with a highly convoluted cerebral cortex [29]. As described above, California sea lions have a very long gestation period of approximately eleven months, which is long for most mammals even with the approximate 90 day diapause that yields an actual post-blastocyst fetal growth period of 242 days. Natural observation indicates that young are born exceptionally precocial [1]. Eyes are open and functional at birth indicating prenatal maturation of the optic lobe and myelination of the optic tract. Newborns are capable of highly coordinated motor activities 10 to 15 minutes after birth and able to walk within 30 minutes, indicative of prenatal maturation of the cerebellum, typically one of the last brain regions to develop in mammals. At 2 to 3 weeks of age, pups have formed social groups of up to 200 individuals, a trait reflecting maturation of sensory and motor cerebral modalities. Hence, the California sea lion gives birth to neurologically mature pups, indicating that substantial neurodevelopment is completed *in utero*.

**Figure 5:**
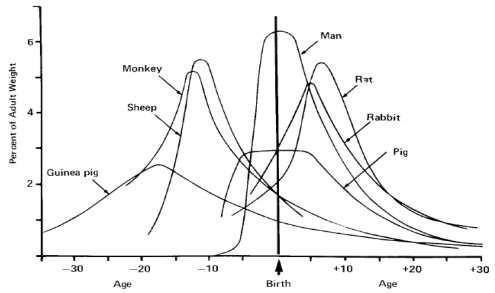
The Brain Growth Spurt of Seven Mammalian Species Growth was determined by velocity curves of growth for each species during pre- and postnatal time frames. The time axis originates from birth with time compressed or expanded relative the length of gestation. Figure is reproduced from reference [31].

Analysis of mammalian brain growth has revealed that growth bursts occur at different times relative to birth for precocial and altricial species [30]. The timing of the brain growth burst relative to the time of birth has been used as a widely accepted measure to evaluate species as prenatal, perinatal or postnatal developers and guide selection of comparative species for neurodevelopment studies (Figure 5) [31].

**Figure 6:**
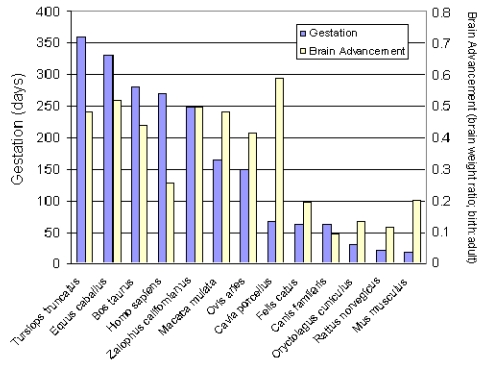
Length of Gestation and Brain Advancement for Thirteen Mammalian Species Brain advancement was determined by the fractional weight of the brain at birth relative to the weight of brain for the adult. The figure is prepared from appendix data of reference [32].

A compilation and analysis of gestation time and brain birth weight was conducted for 91 mammalian species, including the California sea lion, which provides data to better analyze their comparative neurodevelopment [32]. Analysis of this data set for time of gestation and brain advancement at birth (i.e. brain weight birth/brain weight adult) indicate that the California sea lion is clearly among the prenatal developers based on timing of the brain growth burst (Figure 6). This places neurodevelopment of the California sea lion along laboratory and domestic species, in between the guinea pig and horse, which have a higher index, and the cow, rhesus macaque and sheep, which have a lower index. Other, more commonly investigated species, however, such as the human, domestic cat, dog, rabbit, laboratory mouse and rat, have a substantially lower index for prenatal brain development; therefore, these species are not optimal for direct comparison of *in utero* neurodevelopment with sea lions. Among the precocial species with experimental neurodevelopment data relevant to interpolation of domoic acid toxicity in California sea lions are the rhesus macaque (*Macaca mulata*) and domestic sheep (*Ovis aries*), and each are used for further analysis in this review. These two species have a somewhat shorter gestation period and smaller brain size; yet, their comparable degree of *in utero* brain growth remains a valuable basis for comparison with the California sea lion.

**Figure 7:**
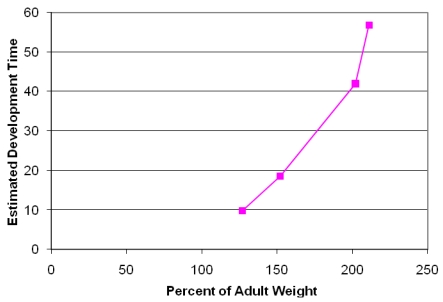
Brain Advancement for Four Fetuses Collected from Stranded California Sea Lions Brain advancement was determined by the fractional weight of the brain at birth relative to the weight of brain for the adult. Embryonic development was determined as the time of diapause to time of collection of stranded California sea lion plus six days development to blastocyst stage. Diapause was determined as date corresponding to photoperiod of 11.48 h/day for the Channel Island rookeries.

Analysis of fixed brain weight from the four collected prenates at E127, E152, E202 and E211 provides age dependent data that this species clearly is a prenatal developer with a brain growth burst between approximately E150 and E200 (Figure 7). The synchronization of diapause, permits an estimate of developmental age of the fetuses using the date corresponding to photoperiod of 11.48 h/day for the Channel Island rookeries as day 6 of development and the collection date of the stranded sea lion as the last day of development. Our data indicates that fetal brain weight relative to adult weight is advanced further than that estimated from Sacher and Staffeldt [32], likely reaching a highly precocious 60% or greater brain advancement index.

**Figure 8:**
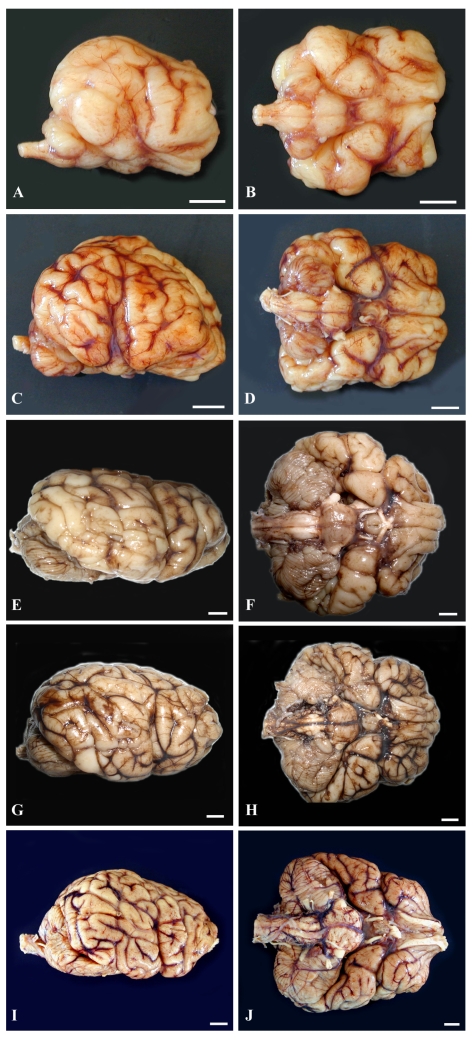
Comparative Lateral and Ventral Gross Views of Successive Prenatal California Sea Lion Brains and an Adult Brain (ruler: 1 cm). Based on a diel estimation of release from embryonic diapause, these animals’ embryonic age is estimated **(a–b)** E127, **(c–d)** E152, **(e–f)** E202 and **(g–h)** E211 days and then **(i–j)** adulthood. Images demonstrate the variable progression in development of the brainstem, cerebellum and cerebrum, nearing adult development by E211. (Photographs g–h courtesy of Elizabeth Wheeler at TMMC).

The lateral and ventral gross views of the formalin-fixed brain from the four prenates are shown in Figure 8 to help demonstrate brain development and the growth burst of three major regions, the brainstem, cerebellum and cerebrum.

**Figure 9:**
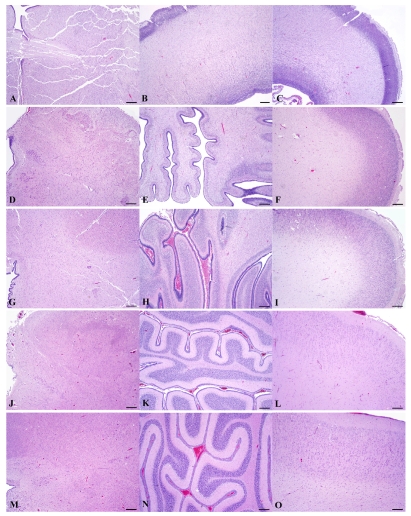
Comparative Histomorphology of Brainstem, Cerebellum and Cerebrum (Parietal Lobe) of Successive Prenatal California sea lion Brains (hematoxylin-eosin stain; rulers 300 μm). Based on a diel estimation of release from embryonic diapause, these animals’ post-blastocyst time of development is approximately **(a–c)** E78, **(d–f)** E127, **(g–i)** E152, **(j–l)** E202 and **(m–o)** E211 days. Photomicrographs are provided to elucidate the development in gross anatomy (Figure 8) by demonstrating a corresponding variable progression in neural organization in each region, nearing adult development by E211.

Histomorphology of each of the three regions from age-matched prenates and an earlier prenate (E78) are provided for further elucidation of the gross anatomy (Figure 9). Histomorphology of the prenate at E78 shows non-distinct neural organization in all regions. Grossly, the prenate at E127 has a brainstem that is more developed than the cerebellum or cerebrum with defined regions that still are less pronounced than at E152, which corresponds to histomorphology showing greater progression in neural organization in the brainstem than in the other regions. The cerebellum is proportionately small, especially the vermis (median elevation), and has relatively poor definition of folia. The cerebrum has little gyral formation. Grossly, the prenate at E152, at the beginning of the growth burst, shows slight increased development in the brainstem but the most notable change is in the cerebrum and the cerebrum and to a slightly lesser extent the cerebellum, which corresponds to the progression in neural organization in each region shown by histomorphology. The cerebrum has significantly increased gyral formation, and the cerebellum is proportionately larger, more defined folia but the vermis remains the least developed region. Grossly, the prenate at E202, near the completion of the growth burst, shows a similar gradual increase in brainstem development, and a most notable progression in the cerebellum and to a lesser extent the cerebrum, which corresponds to the progression in neural organization in each region shown by histomorphology. The cerebellum again is proportionately larger, almost to the proportion of the adult but with the vermis still remaining underdeveloped. The cerebral gyrus formation is nearing that of the adult, but is not as markedly different from E152 as compared to the transition from E127 to E152; however, by histology, the neural organization seems to have progressed more than from E127 to E152. Grossly, the prenate at E211, near the completion of the growth burst, shows a similar gradual increase in development in all regions resulting in anatomy that nears that of the adult, which again corresponds to the progression in neural organization in each region shown by histomorphology.

All developmental toxicity of domoic acid reported in the literature to date has been conducted in mice and rats, which are both altricial species utilized extensively as comparative species for human neurodevelopment [33]. Accordingly, consideration of the only experimental domoic toxicity data sets requires interpolation of data from these altricial species that do not correspond well to the precocial neurodevelopment timeline of the California sea lion. Thus, comparisons are best accomplished through the use of allometric models, which is founded on the widely accepted assumption of a common brain map for development and comparison of developmental markers from model species [34]. Accordingly, we subsequently describe three forms of domoic acid-induced symptoms occurring at different developmental times in the rat and then use allometric methods to align these findings to the developmental times of the California sea lion.

## Domoic Acid Induced Neurogenesis/Neuronal Migration Toxicity in Mice and Rats

Neurogenesis is an early event in brain development and one that follows a precise pattern highly conserved between mammalian species. It begins with the folding of the neurotube and proceeds in a process described by the prosomere hypothesis, which proceeds from spinal cord to telenocephalon with an added lateral direction, such that the most late forming areas are the anterior lateral parts of the brain including the hippocampus, neocortex and olfactory bulb [35]. The length of time for neurogenesis is related to the ultimate size of the brain or specific brain region indicating that allometric methods can predict the timing of neurogenesis in different species [36]. Early in neurogenesis, neuroepithelial cells give rise to radial glial cells, which are progenitor cells possessing neuroepithelial and glial cell properties that serve to physically support and guide migration of neurons, as well as continue to give rise to new neurons [37,38]. From a toxicological perspective, this makes dissociation of toxicant effects on neurogenesis and migration difficult to discern precisely; accordingly, neurogenesis toxicity and neuronal migration toxicity are best viewed together.

**Figure 10:**
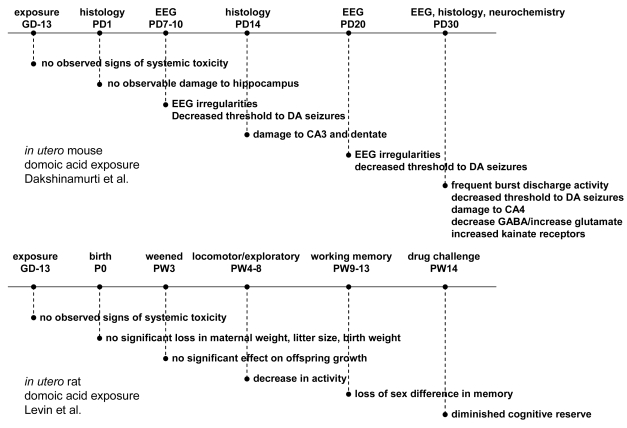
Timeline for Experimental Findings of Two Studies on the Effect of Domoic Acid at Gestational Day 13 Top panel (A) shows timeline for exposure of mice at GD13 and results from histology, EEG and neurochemistry at later postnatal times [39]. Bottom panel (B) shows timeline for exposure of rats at GD13 and results from neurobehavioral testing [41].

A seminal study for domoic acid toxicity during the prenatal period was conducted in gestational day (GD)13 mice (Figure 10a) [39]. No observational effects were noted in the dams treated intravenously with 0.6 mg/kg domoic acid at the time of treatment or the neonates at birth. The postnatal animals, however, showed increasing irregularities in electroencephalogram (EEG) recordings accompanied by a reduced threshold to domoic acid-induced seizures that intensified from mid to late postnatal life. Altered morphology of the hippocampus was not evident on the first postnatal day, yet began to appear midway through the postnatal period (PND 14), a time corresponding to the brain growth burst in rats and mice. The altered morphology of the hippocampus resulting from the GD13 domoic acid exposures was associated with a neurochemical basis for excitability, as indicated by an increased neurotransmitter ratio of glutamate:GABA in the brain and an increased density for domoic acid receptors, as assessed by kainic acid binding sites. The exposure to domoic acid at GD13 corresponds to the beginning of neurogenesis in the hippocampus and this suggests that toxic action on neuroprogenitor cells may lead to an altered neuronal or glial circuitry organization that promotes an excitatory (glutamatergic) over inhibitory (GABAergic) balance in the hippocampus. Analogous effects also have been reported after *in utero* administration of the mitotic inhibitor methylazoxymethanol to rats [40] consistent with an action to inhibit clonal expansion of neuroprogenitor cells. The adverse effects of early developmental exposure to domoic acid also include effects on memory and learning, as determined in the offspring of rats treated subcutaneously with 1.2 mg/kg with domoic acid at GD13 (Figure 10b) [41]. Domoic acid led to a normalization of sex based differences that occur in radial-arm maze testing. Additionally, when the rats were exposed to the amnesic drug scopolamine, animals exposed to domoic acid *in utero* showed significant impairment in response time. These results indicate that exposure to domoic acid during neurogenesis decreases the cognitive reserve as the animals mature to juveniles. The linkage of the toxic effects of domoic acid to seizure activity and memory likely result from the sharing of common pathways gated by the targeted dentate granule cells of the hippocampus, which may predispose animals to behavioral dysfunction and epilepsy [23].

## Domoic Acid Induced Seizure Behavior in Mice and Rats

Early in postnatal life mice and rats are highly sensitive to domoic acid induced seizures [42] The manifestation of postnatal domoic acid-induced seizure behavior in rats progresses from continuous tonic-clonic convulsions to adult-like full limbic seizure behaviors, including head nodding, mastigation, salivation, paw clasping and fine generalized tremors [43,44]. EEG analysis of postnatal mice exposed intravenously to 0.6 mg/kg to domoic acid reveals only occasional spiking activity at PND10 and a progression from intermittent spike activity to burst discharge activity by PND30 [39]. Difficulty in recording early prenatal brains, however, limits tracing propagation of epileptiform activity in the immature limbic system. In the adult rat, EEG analysis indicates that domoic acid causes progressive epileptiform activity that spreads from the hippocampus to the sensiomortor cortex achieving limbic motor status eventually leading to generalized status epilepticus and tonic seizures [45]. Extensive analysis of postnatal seizure behavior in the rat has been analyzed for the related neurotoxin kainic acid. Although subtle differences are noted for kainic and domoic acid-induced seizure behaviors [43], the kainic acid data are essential to understand the development of seizure behavior.

Early postnatal rats show tonic or tonic-clonic convulsions to kainic acid, yet limbic motor seizures are not evident until PND21. Metabolic mapping indicates that the action of kainic acid in these mice is limited to the CA3 region of the hippocampus (note that the dentate granule cells and mossy fibers have not yet matured) and lateral septum from PND3 to 21 and extends to other regions of the limbic brain after this time [46]. Similarly, cellular damage is observed in interneurons and not pyramidal cells [47]. This pattern is consistent with maturation of connections within the hippocampus and to other limbic regions, particularly the amygdala occurring at PND21, appearance of kainic acid sensitive ionotropic glutamate receptors and the establishment of kainic acid induction of c-fos expression in limbic structures [47–49]. Although the long term consequences of postnatal seizures on predisposition to epilepsy is debated, postnatal seizures from even a single postnatal exposure to kainic acid impairs spatial memory tasks [50]. Even a single kainic acid induced seizure at P7 can impair glutamaterigic synapses and performance in memory tasks in adult animals [51]. Permanent effects of postnatal kainic acid have been replicated with domoic acid exposure during the PND 8–12 window resulting in seizure behavior and decreased performance in spatial memory tasks of adult rats [52].

## Allometric Interpolation of Developmental Toxicity from Rats to California Sea Lions

**Figure 11:**
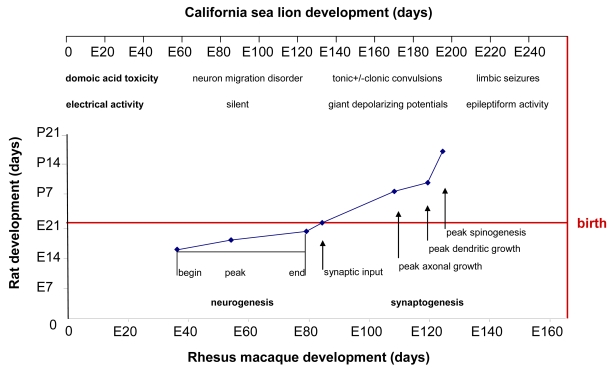
Correlation of Rat and Rhesus Monkey Neurodevelopment with Gestational Scaling to the California Sea Lion Data from milestones of neurodevelopment in developing rat and monkey limbic brains is plotted from [34]. Scaling of California sea lion post-implantation gestation relative to monkey gestation is shown on top of chart. Overlay of hippocampal neurogenesis milestones is from reference [53]. Overlay of brain growth spurt is from reference [31]. The domoic acid toxicity and electrical activity time line are modified from rhesus macaque neurodevelopment [54] and scaled to the California sea lion.

A detailed allometric analysis of neurogenesis and other developmental milestone events in various brain regions in eight commonly investigated mammalian species has yielded relationships by which to compare the timing of neurodevelopment [34]. Rat was the most extensively documented species, and rhesus macaque was the only precocial species in this analysis. The modeling of data for rat and macaque yielded a near linear relationship for the first 117 days of limbic development of the macaque. We have used a similar comparison of the seven milestone events in hippocampal development in the rat and macaque. We then applied a comparative overlay of the development of the California sea lion to the macaque, based upon their similar degree of brain advancement at birth, to yield a comparable relationship of developmental toxicity of domoic acid between the rat and the sea lion (Figure 11).

**Figure 12:**
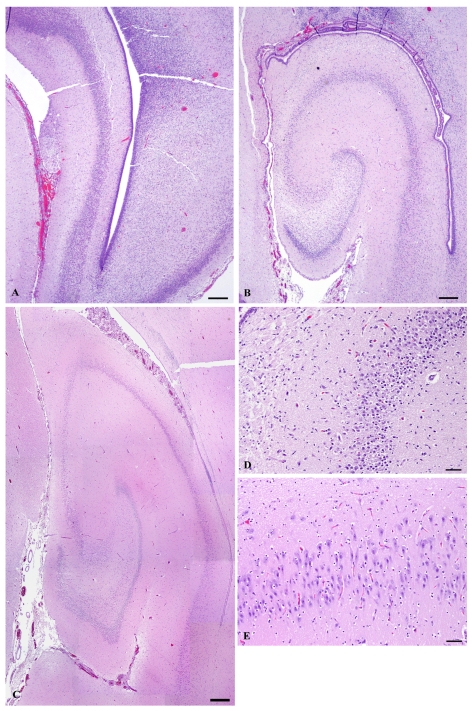
Comparative Histomorphology of the Hippocampus from Successive Prenatal California Sea Lion Brains (hematoxylin-eosin stain: rulers (a–c) 300 micrometers, (d–e) 50 micrometers). Based on diel estimation of release from embryonic diapause, these animals’ post-blastocyst time of development is approximately **(a)** E78, **(b, d)** E127, and **(c,e)** E202. Photomicrographs demonstrate that neurogenesis of the hippocampus is complete during the early second trimester in the first half of embryonic development, as there is no neural organization near the end of the 1st trimester at E78 **(a)** versus the presence of neural organization in the cornu ammonis **(b, d)** and dentate gyrus **(b)** regions at E127. Comparatively at E202, neural organization is more refined in the cornu ammonis **(c, e)** and dentate gyrus **(c)**, a time following peak synaptogenesis.

In the rat, neurogenesis of the pyramidal cells of the hippocampus occurs during the last third of the prenatal period, estimated to conclude at E20 [55], whereas in the rhesus macaque it occurs during first half of the prenatal period, estimated to conclude at E80, just prior to midpoint of embryonic development [53]. Although neurogenesis usually is determined by methods measuring cell division, histomorphology of the hippocampus from the E78 prenate showed a lack of neural organization (Figure 12a) and that from the E127 prenate showed that pyramidal cell layers were formed (Figure 12b&d). This progression indicates that neurogenesis of the hippocampus is largely completed in the first half of embryonic development in the California sea lion. It is during this developmental period that brain electrical activity is silent, as the pyramidal cells have not formed synapses. Nonetheless, domoic acid has toxic outcomes as described above, likely resulting from disruption of the migration of radial glial cells to the hippocampus leading to alteration of GABAergic hippocampal interneurons or glutamatergic astrocytes in the pyramidal cell layer [56,57].

At the time of parturition in the rat and the midpoint of *in utero* development in the macaque, hippocampal pyramidal cells begin to receive primitive excitatory GABAergic synapses from interneurons. The following ten days of postnatal development of the rat and the third quarter of embryonic development of the macaque are characterized by differentiation of hippocampal pyramidal cells, corresponding with the brain growth burst. This process is evident morphologically by axonal growth and formation small apical dendrites. Morphometric studies in rat and macaque indicated that the first of these two milestones occur for axonal growth at P7 in rat and E109 in macaque and for dendrite formation at P9 in rat and E120 in macaque [54]. Functionally, this timeframe corresponds with synchronized hippocampal discharges referred to as giant depolarizing potentials (GDPs). GDPs are slowly propagating waves that commonly originate in the CA3 field of the hippocampus and propagate via CA1 bilaterally to the hippocampi and septa [58]. Of the collected California sea lion prenates, the brain of E152 corresponds to this stage of transition from “silent” neurons to those with primitive patterns of synchronized activity. It is during this mid prenatal period of the California sea lion developmental period that domoic acid has the potential to cause tonic or tonic-clonic convulsions.

The second ten days of postnatal development in the rat and the final quarter of embryonic development in the macaque are characterized by the acquisition of dendritic spines by hippocampal pyradimal cells. Morphometric studies in rat and macaque indicated that spinogenesis peaks at P16 in rat and E125 in macaque [54]. Functionally, this timeframe corresponds with transition from GDPs to increasing epileptiform activity. Adult-like full limbic discharges can be induced at this developmental window by neuroexcitatory agents such as ionotropic glutamatergic agonists and GABAergic antagonists. Biochemically-induced epileptiform activity corresponds to formation of excitatory glutamatergic synapses and the transition of GABAergic synapses from excitatory to inhibitory [58]. Of the California sea lion prenates collected, the hippocampus of E202 (Figure 12c&e) corresponds to this stage of transition from neurons showing primitive patterns of synchronized activity to those with integrated electrical signaling. It is during this late prenatal period of the California sea lion that domoic acid has the potential to cause full limbic seizures.

## Birth Serves as a Transition Point for Exposure Susceptibility

The length of gestation has its most prominent effect on the route of exposure for charged polar molecules, such as domoic acid, in developing animals. Parturition marks the transition in the primary routes of maternal-neonate exposure, i.e. from vascular transfer via the placenta in the prenatal animal to oral absorption via milk in the postnatal animal. Parturition also marks the transition in elimination, i.e. from a state of minimal functional elimination due to recycling of the toxin between the body and amniotic fluid in the prenatal animal to elimination via urine formation and voiding in the postnatal animal. Finally, brain maturation is associated with restricted movement of charged polar molecules across the blood brain barrier, which decreases susceptibility to neurodevelopmental damage as animals mature.

Analysis of exposure in developmental toxicity is essential because of its preponderance to accentuate sensitivity of the early developing nervous system to toxicants. Exposure susceptibility of neonatal animals to domoic acid only has been analyzed in rats. Although rats provide a good comparative model for placental transfer in sea lions, rats do not provide as good of a comparative model for re-exposure from amniotic fluid and decreased brain permeability, because birth occurs at a much earlier developmental stage for the rat. These factors are evaluated best in precocious species, thus requiring the use of most notably the fetal lamb, supplemented with observational and opportunistic data from the California sea lion.

## Interpolation of Rat Prenatal Exposure Susceptibility to Domoic Acid to California Sea Lions

In the rat, maternal plasma domoic acid readily enters the early and late prenate. After one hour, 5.2 % of maternal plasma concentration accumulates in the amniotic fluid at GD 13 [59]. Maternal to fetal transfer in the rat provides a good animal model for the California sea lion. The sea lion has a zonal endotheliochorial placenta, which is more typical of carnivores except for highly attenuated maternal endothelial cells that yield thin-walled sinusoids [60]. Thus, the sea lion maternal blood is partitioned by very thin-walled (1 micron) sinusoids in comparison to the rat maternal blood that directly bathes fetal membranes. The maternal to fetal placental barrier, however, results more from the fetal membranes than from the sinusoids. Fetal membranes are similar in composition between the sea lion and rat, of which the former has a porous cytotrophoblast and single syncytiotrophoblast cell layer and the latter has a porous cytotrophoblast and double syncytiotrophoblast cell layer [61].

The amniotic fluid is largely in equilibrium with fetal tissues during the duration of gestation, for which the primary route of transfer is transdermal absorption through the unkeratinized skin but also through ingestion. [62]. Although elimination rates for domoic acid from amniotic fluid are not known, analysis of other polar compounds with similar plasma elimination rates suggest that domoic acid will not eliminate rapidly from the amniotic fluid as it does from maternal plasma [59]. This hypothesis is supported by tightly correlated experimental data in the rat [59] and opportunistic data in the California sea lion [12], as domoic acid levels in rat amniotic fluid (8–21 ng/mL) are within the same range of those measured from stranded pregnant sea lions (4–34 ng/mL). Interestingly, domoic acid levels in sea lions were measured as late as eight days after stranding, which suggests that amniotic fluid acts as a sink for domoic acid with the potential to continuously expose the fetus long after the toxin has been cleared from the dam. This source of secondary exposure to prenates likely has a highly significant effect, as reduced domoic acid clearance is a major factor to the higher toxicity observed in immature rats [42].

*In utero* exposure to domoic acid in amniotic fluid may occur both by dermal and gastrointestinal absorption, as aforementioned. Early in gestation, the fetus will be exposed to toxin continuously until the skin is keratinized. In humans, skin keratinization does not occur until the third trimester; however, skin embryogenesis advances beyond this stage in California sea lions. Analysis of skin and hair development in the Steller sea lion (*Eumetopias jubatus*), which shares a similar gestation pattern to the sea lion, indicates that *in utero* skin development is complex [63]. The embryonic rudimentary skin develops to the provisional fetal skin cover (comparable to late gestation skin of terrestrial mammals), and then is replaced by two *in utero* molts. The molt to primary hair and then to the infantile hair each disrupt the outer layers of the epidermis and likely enhances dermal exposure. Yet even in the fetal sheep, which develops wool coats in the last third of gestation, amniotic fluid circulates across the skin until late in development [64]. A substantial decrease in skin permeability *in utero* is believed to occur with the embedding of lipids in the stratum corneum, a process that requires the surge of glucocorticoids that happens at the end of gestation [65]. Accordingly, in California sea lion prenates, high dermal exposure to domoic acid in amniotic fluid is likely during the interuterine period. Later in gestation, amniotic fluid also is absorbed by swallowing. In California sea lion fetuses (estimated GD 201 to 217) from the 2002 standing event, domoic acid was detected in comparable concentrations in fetal gastric fluid and amniotic fluid supporting this later route of sustained exposure even in the late term fetus [12].

## Interpolation of Rat Postnatal Exposure Susceptibility to Domoic Acid to California Sea Lions

Maternal plasma domoic acid also readily enters the milk, posing a potential hazard during the lactation period. Analysis of domoic acid transfer during lactation indicates that milk concentrations were 6% of the one hour maternal plasma levels [66]. The efficiency of lactational transfer of domoic acid to postnatal rats was determined by oral gavage of rat milk and of milk collected from domoic acid treated lactating dams. The results of this study, as determined by monitoring domoic acid blood levels in the postnatal rats, indicated that the amount of domoic acid to which a neonate may be exposed following a single feeding from a dam receiving a low symptomatic dose is three orders of magnitude less than the oral dose causing observable symptoms in neonates. Hence, birth indeed is a transition of exposure susceptibility to domoic acid.

## Interpolation of Fetal Rat and Sheep Brain Exposure Susceptibility to California Sea Lions

The previous discussion provides evidence that prenatal California sea lions are substantially more susceptible to domoic acid exposure than animals later in life. Considering the long duration of gestation in the California sea lion, the substantial degree of neurodevelopment occurring during this time and the potential for *in utero* transdermal absorption, a prolonged window of susceptibility for neurotoxicity is expected. An additional factor influencing the susceptibility of the developing California sea lion to domoic toxicity is brain permeation to domoic acid, a 331 g/mol (Dalton) water-soluble acidic amino acid with three carboxylic acids, which at physiological pH predominantly is deprotonated at all three carboxyl groups and protonated at the amino group yielding a net charge of negative 2 [23].

In the developing brain of rats, the total protein in the cerebral spinal fluid is equivalent to that in the amniotic fluid at approximately E11.5, most likely because the amniotic fluid is trapped within the neural tube on closing of the anterior neuropore [69]. Thereafter, with the development of the ventricles and its choroid plexus with the tight junctions of the blood-brain barrier, which proceeds in a parallel timeline with the growth of the brain, the concentrations between the two regions begin to diverge and total protein declines. The developing brain as compared to adults, however, remains more permeable to proteins, especially endogenous proteins, as well as to small polar molecules most likely due to selective transcellular transfer mechanisms in the developing choroid plexus epithelial cells rather than due to an immature blood-brain barrier as presumed previously. The role of selective transport mechanisms across the endothelial cells of the blood-brain barrier is not known for prenates like it is for adults [67]. Subsequent to entry into the cerebral spinal fluid, small polar molecules will redistribute into the extracellular space of the brain. Similar to the aforementioned trends, *in utero* exposure to domoic acid results in a concentration equilibrium between the brain and amniotic fluid of the prenate rat at E13 and declines by half in the brain at E20 [59]. Whether the same selective transport mechanisms post-closure of the anterior neuropore are used for domoic acid as for the aforementioned proteins and small molecules is not certain, because in the adult brain, the exogenous domoic acid enters at a lower transfer rate seemingly by a different mechanism than its endogenous analogue L-glutamate [67].

Brain permeability does not decrease substantially in the altricial developing rat until shortly after birth. As the California sea lion is neurologically more mature at birth, evidence from precociously developing species is necessary to develop a time frame for restriction mechanisms that limit brain exposure to domoic acid. The fetal lamb is the best studied precocial animal species for the development of the blood-brain barrier and accordingly provides the best timeline comparison for the California sea lion. Studies in GD50 fetal sheep indicate that small polar molecules accumulate rapidly in the brain and cerebral spinal fluid, but this permeability declines precipitously between GD 60 and 70 to reach half of adult levels. After GD70 in the fetal sheep, permeability continues to decline to adult levels, which are reached by GD123 [68]. Factors of brain maturity at birth (0.42 adult brain weights at birth) and time of gestation (150 days) for the sheep project to maturation of the blood-brain barrier at GD89, reaching half adult levels by GD103 and reaching adult permeability by GD176 in the California sea lion. Hence, although exposed by placental transfer, the fetal sea lion brain likely is freely permeable to domoic acid for the first trimester, develops reduced permeability the second trimester and achieves an advanced permeability barrier for the last trimester of gestation (Table 1).

Although the blood-brain barrier is known as a prominent mechanism to protect the brain from many blood-borne hydrophilic compounds, it is overwhelmed readily by domoic acid when the normally efficient renal clearance of domoic acid is compromised. Renal compromise has been proposed to be a predisposing factor to toxicity in immature and aged rats [42,69,70]. Additionally, renal clearance inhibitors in adult rats have been shown to increase radio-labeled domoic acid entry into the brain [71]. Hence, even in situations where the blood-brain barrier is fully functional, it is overcome more when domoic acid is not eliminated efficiently from the plasma. Pregnancy represents such a situation due to poor fetal clearance of plasma domoic acid as well as reabsorption of domoic acid from amniotic fluid.

## Timing of Diapause Forecasts Fetal Toxicity of the California sea lion

**Figure 13:**
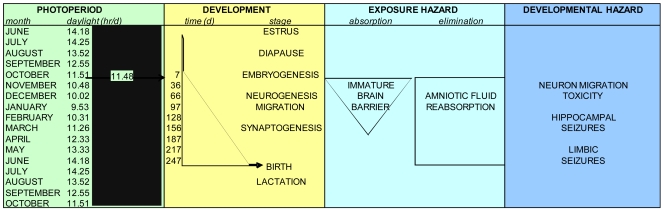
Scaling of Photoperiod, Neurodevelopment, Exposure Hazard and Developmental Toxicity to Months of Year at Channel Island Rookeries Photoperiod is determined as number of day light hours at coordinates 120°24′ W, 34°20′ N from the San Miguel Rookery using U.S. Naval Observatory data and is shown as the curvilinear plot on the left panel. Neurodevelopment milestones are taken from Figure 7 and are synchronized to release diapause at 11.48 hour/day. Exposure hazard and developmental toxicity are scaled from analysis described in the text.

Female California sea lions are a group that is highly susceptible to domoic acid toxicity due to year-round foraging near rookeries that have become increasingly subject to domoic acid-producing diatom blooms. The susceptibility of this population is reflected in their 24:1 female: male sex predisposition to domoic acid toxicity [72]. The eleven month pregnancy of sea lions results in a high risk for prenatal toxicity. To date, experimental research of domoic acid toxicity during development has been conduced in rats and mice, both altricial species not directly relevant to the precocial sea lion. This review has described a rational for use of allometric methods to develop a neurodevelopmental timeline for California sea lions. These allometric projections indicate that the sea lion prenates likely will develop neuron migration toxicity with domoic acid exposures between E50-120, hippocampal seizures with exposures between E120-200 and limbic seizures with exposures after E200. California sea lion mortality events associated with domoic acid toxicity have been analyzed retrospectively from 1991 to 2000 and were found to predominate between May and October [72]. These events corresponded to the last trimester of pregnancy and during the weaning of previous year pups. The reproductive toxicity observed in stranded adult animals included premature live births, sometimes associated with uterine lesions, death *in utero* and induced or naturally occurring abortion, and in the fetus was associated with generalized brain edema and placental abruption [10,12,13,79]. *Pseudo-nitzschia* blooms with substantial toxicity conveyed into the food web, however, also have occurred in late fall [73–75] and early winter [76,77], which would translate to effects in the early to mid prenatal period. The greater exposure susceptibility of the prenatal sea lions also indicates that prenatal toxicity may occur with domoic poisoning that is sub-lethal or non-symptomatic in the adults and hence at times of the year not usually noted for California sea lion acute toxicity events, as has been associated with some premature pupping and mortality of first to third trimester fetuses [79]. This analysis provides a reference by which to forecast potential fetal toxicity of California sea lion by the monthly occurrence of toxic *Pseudo-nitzschia* blooms and the transmission of domoic acid through the food web (Figure 13).

## A Fetal Basis to Adult Disease in the California Sea Lion

Early in development, during the proliferation and migration of hippocampal interneurons and pyramidal cells, the embryonic brain is electrically silent; accordingly, the effect of domoic acid is also silent. Domoic acid poisoning during this time frame becomes apparent only later in life with effects such as increased susceptibility to seizures and decreased memory reserve [39,41]. These effects are consistent with abnormal organization of glutamatergic and GABAergic synapses in the hippocampus as result of disruption of proliferation and/or migration of hippocampal interneurons; a process referred to neuronal migration disorder [40]. Later in development, upon the beginning of formation of synapses and the maturation of axons and dendrites, the electrical activity of the hippocampus begins with slowly developing and transmitting synchronistic discharges, which are believed to guide and strengthen the thousands of connections between pyramidal cells and interneurons [58]. Domoic acid poisoning during this time frame is observable as immature seizure behaviors [43]; however, the long-term results of domoic acid on the brain during synaptogenesis also may lead to increased excitability later in life and to abnormal glutamatergic - GABAergic circuitry [78]. A milestone in excitability during the end of synaptogenesis is the conversion of GABAergic synapses from excitatory to inhibitory as a result of maturation of chloride ion transport. This corresponds to a transition from GDPs to epileptiform activity and more mature seizure behavior resulting from spread of activity throughout the limbic brain and other brain centers [79]. Exposure of rodents to domoic acid or kainic acid during this time frame causes full limbic seizures [43] which can also lead to increased excitability later in life [80]. The cause of excitability once again implicates abnormal glutamatergic - GABAergic circuitry, although involving different mechanisms [80]. For example, analyses of hippocampal pathology in patients with human temporal lobe epilepsy first diagnosed in childhood indicates several characteristic pathological bases, including changes outside and within the hippocampus [81]. The individuals with hippocampal sclerosis showed both a physical and functional loss of GABAergic interneurons. In fact, one case of human domoic acid exposure in a 79 year old male was found three years later to lead to epilepsy [82]. Several hypotheses by which a prior domoic exposure can predispose an individual to epilepsy have been proposed including the pruning of synapses after domoic acid exposure and subsequent sprouting of recurrent collaterals that are glutamatergic rather than GABAergic [23]. Regardless of the mechanism that reduces GABAergic input to the hippocampal pyramidal cells, the functional result is an excitable hippocampus that is less able to control the lateral spreading of electrical discharges across the pyramidal cell layer.

Because the fetal period of the California sea lion encompasses all of the above mentioned periods of brain development, domoic acid exposure at various times of *in utero* development can be expected affect seizure susceptibility later in life. Such effects on the fetus that are manifest later in life are now recognized as the emerging discipline of fetal basis to adult disease, which predicts peak impact in the earlier *in utero* development for increased susceptibility to dysfunction later in life [83]. The zebrafish, is now being applied as an alternative animal model to facilitate analysis of exposure scenarios involving different times of exposure and multiple agents relevant to the California sea lion. The zebrafish shares remarkable commonality with the common rat and mouse models in terms of neurogenesis and migration, development of GABAergic and glutamatergic neurotransmitter systems, spatial cognitive skills, and seizure behavior in response to GABAergic and glutamatergic agents including domoic acid. [40,84–86]. In one study, animals were exposed to domoic acid during neurogenesis and synaptogenesis and then acutely with the GABA A antagonist pentylenetetrazole, shortly after brain maturation [87]. This study determined that exposure to domoic acid during neurodevelopment, resulted in nonsymptomatic animals with increased seizure response, both in terms of time to develop seizures and severity of seizures [87]. More recently, the zebrafish model has been expanded investigate the interactive effects of DTTs and domoic acid [88]. Zebrafish were exposed to DDT during neurogenesis and synaptogenesis and acutely exposed to domoic acid shortly after brain maturation using an exposure scenario meant to correspond to transfer of maternal organochlorine body burden during the course of fetal sea lion neurodevelopment and exposure to domoic acid late in the *in utero* period. This study demonstrated that exposure to DDTs during neurodevelopment leads to asymptomatic animals with greater sensitivity to domoic acid induced seizures. The effect level for the p,p′-DDE congener was found at a zebrafish body burden that corresponds late term fetal sea lion body burden, indicating that organochlorine load transferred to the fetal California sea lion may enhance domoic acid *in utero* toxicity and yield more complex fetal basis to adult disease in the California sea lion [88].

A fetal basis to adult disease may provide one explanation for the emergence of the novel neurologic presentation of California sea lions suspected to be exposed to domoic acid [79]. In addition to the straight-forward anticipated seizure activity initially associated with acute domoic acid toxicity, there has emerged aberrant behavioral and cognitive activity and a more epileptiform pattern of seizure activity that can develop without re-exposure to domoic acid. This neurologic presentation is more prevalent in juvenile and subadult sea lions and in some cases of behavioral or cognitive dysfunction, is not associated with detectable central nervous system pathology using magnetic resonance imaging, EEGs and histopathology. Considering the histopathology of brain edema in second to third trimester fetuses exposed to domoic acid [10], the high vulnerability of California sea lion prenates to *in utero* domoic acid exposure and the increased excitability of the brain resulting from alterations in neurogenesis, neuromigration and synaptogenesis, we propose that fetal exposure to domoic acid may provide a primary explanation for the novel neurological disease described in the California sea lion population.

## Figures and Tables

**Table 1:** Gestational Timeframe for Exposure Susceptibility of the California sea lion to Domoic Acid. (M-BLOOD) Maternal Blood; (AF) Amniotic Fluid.

GESTATION		EXPOSURE	ABSORPTION	ABSORPTION	BRAIN	NEONATAL
TIME	DAYS	FLUID	1° EXPOSURE	2° EXPOSURE	RESTRICTION	ELIMINATION
EARLY	0–80	M-BLOOD/AF	PLACENTA	DERMAL AF	OPEN	MINIMAL
MID	81–160	M-BLOOD/AF	PLACENTA	DERMAL AF	REDUCED	MINIMAL
LATE	161–242	M-BLOOD/AF	PLACENTA	ORAL AF	COMPLETE	MINIMAL
POST	>242	MILK	GASTROINTESTINAL		COMPLETE	EFFECTIVE
